# Application of Rhamnolipids as Dispersing Agents for the Fabrication of Composite MnO_2_-Carbon Nanotube Electrodes for Supercapacitors

**DOI:** 10.3390/molecules27051659

**Published:** 2022-03-03

**Authors:** Wenjuan Yang, Wenyu Liang, Igor Zhitomirsky

**Affiliations:** Department of Materials Science and Engineering, McMaster University, Hamilton, ON L8S 4L7, Canada; yangw48@mcmaster.ca (W.Y.); liangw26@mcmaster.ca (W.L.)

**Keywords:** manganese oxide, carbon nanotube, composite, supercapacitor, rhamnolipids, dispersant

## Abstract

The high theoretical capacitance of MnO_2_ renders it a promising material for the cathodes of asymmetric supercapacitors. The good dispersion of MnO_2_ and conductive additives in a nanocomposite electrode is a key factor for efficient electrode performance. This article describes, for the first time, the application of rhamnolipids (RL) as efficient natural biosurfactants for the fabrication of nanocomposite MnO_2_-carbon nanotube electrodes for supercapacitors. RL act as co-dispersants for MnO_2_ and carbon nanotubes and facilitate their efficient mixing, which allows for advanced capacitive properties at an active mass of 40 mg cm^−2^ in Na_2_SO_4_ electrolytes. The highest capacitance obtained from the cyclic voltammetry data at a scan rate of 2 mV s^−1^ is 8.10 F cm^−2^ (202.6 F g^−1^). The highest capacitance obtained from the galvanostatic charge–discharge data at a current density of 3 mA cm^−2^ is 8.65 F cm^−2^ (216.16 F g^−1^). The obtained capacitances are higher than the capacitances of MnO_2_-based electrodes of the same active mass reported in the literature. The approach developed in this investigation is simple compared to other techniques used for the fabrication of electrodes with high active mass. It offers advantages of using a biocompatible RL biosurfactant.

## 1. Introduction

The materials science of natural biosurfactants has emerged recently as a solution to introducing new strategies in sustainable manufacturing. Biosurfactants offer numerous benefits, such as low cost, biocompatibility, and biodegradability. Recent reviews highlighted the benefits of bile salts as efficient biosurfactants for solubilization and dispersion of various organic and inorganic materials [1,2]. It was shown that bile salts outperform many commercial surfactants and allow the development of advanced materials and devices for photovoltaic, biomedical, energy storage, and corrosion protection applications [1,2]. The unique functional properties and chemical structure of bile acids and bile salts [3,4,5] make them especially important for the development of biomedical devices and surface modification of biomaterials [6]. The fundamental studies of mussel protein adhesion to different surfaces [7,8] facilitated the development of catecholate biosurfactants with enhanced adsorption on inorganic nanoparticles [9] and new techniques in material processing [10,11].

Rhamnolipids (RLs) are among the most promising biosurfactants for various industrial applications in oil recovery [12], agriculture [13,14], laundry products, and medicine [15]. Due to their unique functional properties, RLs have been utilized for various applications in biomedical field such as antimicrobials, anticancers, immune modulators, and virulence factors [14]. However, the RL potential for the development of functional materials and devices is only beginning to be recognized. Very promising results were obtained in applications of RL as dispersants for BaTiO_3_ particles [16]. RL showed superior BaTiO_3_ particle dispersion compared to other dispersants, such as polyelectrolytes [16]. In addition, RL showed efficient dispersion of alumina [17,18], zirconia [19], and hematite [20] particles in aqueous suspensions. The use of RL as capping agents for chemical synthesis allowed the fabrication of ZnS nanoparticles with controlled sizes in the range of 1–10 nm [21]. The morphology of NiO particles, prepared by an aqueous chemical precipitation method, was influenced by adsorbed RL [22]. Silver nanoparticles were prepared using RL as the capping and stabilizing agent by using chemical [23] and electrochemical synthesis [24]. RLs are very promising for various applications as capping and dispersing agents because their critical micelle concentration is 10–100-times lower than that of traditional chemical surfactants [15]. RLs are non-toxic, biocompatible, chemically stable, and low-cost biosurfactants [15].

This investigation was motivated by unique functional properties of RL, which make them promising dispersants for colloidal nanotechnology. The goal of this investigation was the application of RL for the fabrication of composite MnO_2_-carbon nanotube electrodes for electrochemical supercapacitors. In this approach, carbon nanotubes were used as conductive additives. The capacitance of carbon nanotubes is significantly lower [25] than the capacitance of MnO_2_. Therefore, the use of carbon nanotubes as conductive additives allowed better utilization of capacitive properties of MnO_2_. Multiwalled carbon nanotubes were used due to their lower cost, compared to single-walled carbon nanotubes. The results presented below indicated that RL can be used as efficient dispersants and co-dispersants for MnO_2_ and carbon nanotubes. We discussed adsorption and dispersion mechanisms and linked them to the structure features of the RL molecules. Electrochemical testing results showed the enhanced performance of MnO_2_-carbon nanotube electrodes, which resulted from their advanced microstructure.

## 2. Results and Discussion

**Figure 1:**
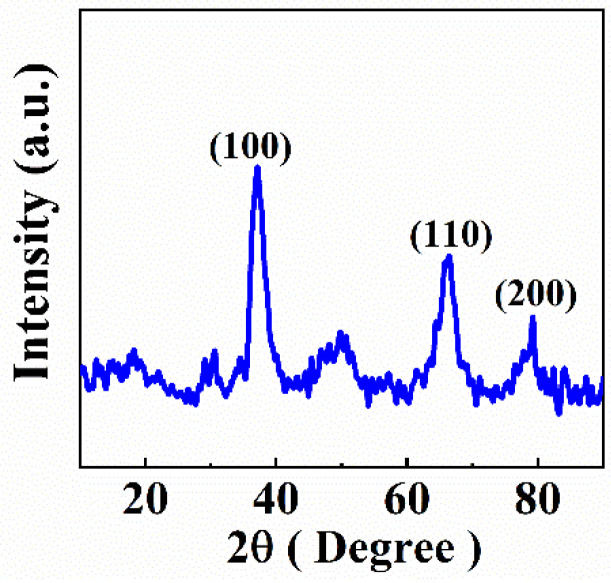
X-ray diffraction pattern of as-prepared MnO_2_.

Figure 1 shows the X-ray diffraction pattern of MnO_2_ prepared in this investigation. The X-ray diffraction studies revealed poor crystallinity of the obtained material. The X-ray diffraction pattern showed small diffraction peaks, which corresponded to ε-MnO_2_ (JCPDS file 030-0820). However, the studied material also contained an amorphous phase.

The charging mechanism of MnO_2_ in the Na_2_SO_4_ electrolyte involves the following electrochemical reaction.

MnO_2_Na ↔ MnO_2_ + e^−^ + Na^+^ (1)

It is observed that good electronic conductivity and electrolyte access to the MnO_2_ material surface are necessary for efficient charge–discharge reactions. However, the electronic conductivity of MnO_2_ is low, and conductive additives, such as MWCNT, are necessary for the fabrication of advanced electrodes [25,26,27]. Electrode microstructure and composition are important factors for the efficient electrochemical performance [28,29,30,31]. Good electrochemical performance must be achieved at high active mass loadings (AML) [32] in order to reduce the contribution of current collectors and other passive components to the total mass of electrodes and devices. The typical AML of commercially activated carbon-based electrodes [32] is 10–20 mg cm^−2^. However, significantly higher AML of about 40 mg cm^−2^ is necessary [32] for the fabrication of electrodes of the same volume and based on inorganic materials with higher density, such as MnO_2_. The commercial Ni-foam current collectors used in this investigation are designed for supercapacitors and batteries based on inorganic charge storage materials, with an AML of 30–50 mg cm^−2^.

It is known that the capacitance of MnO_2_-based electrodes prepared without conductive additives is low [33], typically 0.88 F cm^−2^ (22 F g^−1^) at AML of 40 mg cm^−2^. Higher capacitances can be achieved by using conductive additives, which improve electronic conductivity. The as-received MWCNT used in this investigation consisted of large agglomerates with a typical size of 0.5 mm [34]. The SEM images of such agglomerates were presented in a previous investigation [34]. The mechanical mixture of MnO_2_ and MWCNT showed a higher capacitance of 2.1 F cm^−2^ (52.5 F g^−1^) [35]. It was found that good dispersions of MnO_2_ and MWCNT are necessary for the better utilization of capacitive properties of MnO_2_ and for achieving higher capacitances. Therefore, good dispersants are critically important for enhanced dispersion and mixing of MnO_2_ and MWCNT. The need in advanced dispersants for the development of efficient supercapacitors has generated tremendous interest in the search for advanced dispersant molecules.

**Figure 2:**
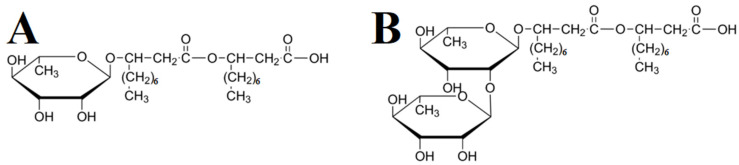
Chemical structures of (**A**) mono-RL and (**B**) di-RL.

In this investigation, RLs were tested as new dispersants for the fabrication of MnO_2_-MWCNT electrodes for supercapacitors. RL biosurfactants are amphiphilic glycolipids produced by Pseudomonas aeruginosa [15]. As received RLs were a mixture of mono-RL and di-RL molecules. The chemical structures of such molecules are presented in Figure 2. The structures contain L-rhamnose and β-hydroxyl fatty acids [15]. The amphiphilic structure of RL and electric charge of their carboxylic groups in solutions are important factors, which make RL promising dispersants for the electrostatic dispersion of materials.

**Figure 3:**
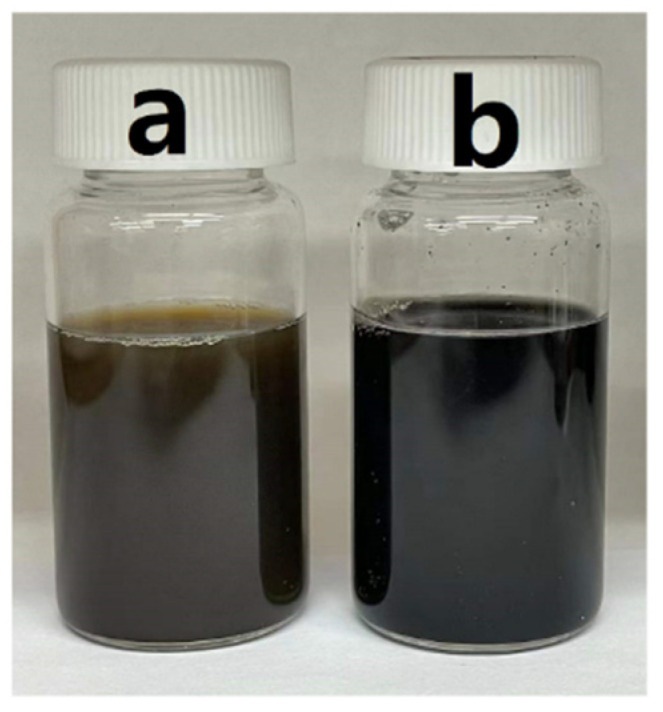
Suspensions of (**a**) MnO_2_ and (**b**) MWCNT, containing RL as dispersants 7 days after preparation; mass ratio of RL:MnO_2_ and RL:MWCNT is 0.2.

Sedimentation tests showed that RL allowed good dispersion of both MnO_2_ and MWCNT (Figure 3).

It was hypothesized that the amphiphilic chemical structure of RL was beneficial for adsorption on both MnO_2_ and MWCNT, and the negative charge of the dissociated COO^−^ groups facilitated electrostatic dispersion. It is known that fatty-acid type surfactants containing hydrophobic hydrocarbon groups provide good dispersion of carbon nanotubes [2,36,37]. The adsorption of such surfactants on carbon nanotubes is based on hydrophobic interactions of the hydrocarbon groups of the surfactants with side walls of carbon nanotubes. Therefore, the fatty acid groups of RL are beneficial for their adsorption on MWCNT and MWCNT dispersion. Previous studies showed that RLs exhibit strong chelating properties and are used for the removal of metal ions from solutions [38,39]. Therefore, the chelation of Mn atoms on the surface of MnO_2_ particles can promote RL adsorption. It is known that chelating groups and OH groups of dispersants are beneficial for their adsorption on inorganic nanoparticles and the dispersion of nanoparticles [9].

**Figure 4:**
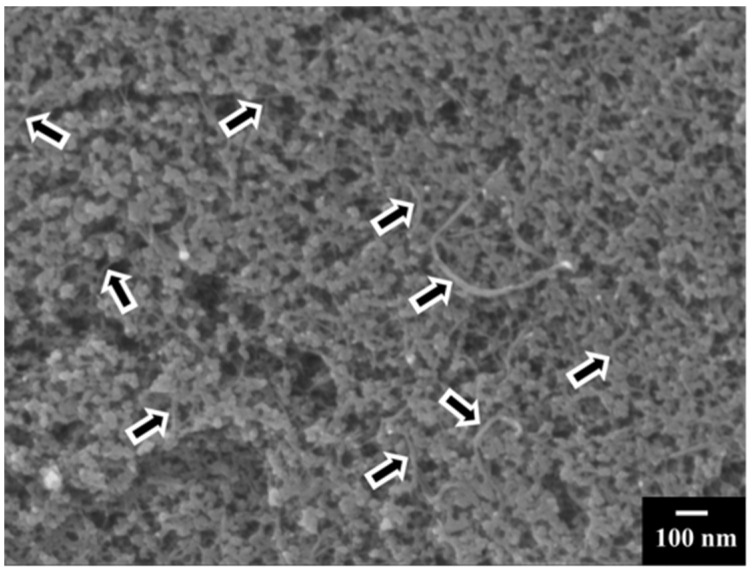
SEM image of MnO_2_-MWCNT electrode. Arrows show MWCNT.

Figure 4 presents a typical SEM image of a MnO_2_-MWCNT electrode prepared using RL. The SEM studies of the composite electrode showed nonagglomerated MnO_2_ nanoparticles and MWCNT distributed between the nanoparticles. The SEM studies confirmed good mixing of MnO_2_ and MWCNT, which resulted in the enhanced performance of the electrodes.

**Figure 5:**
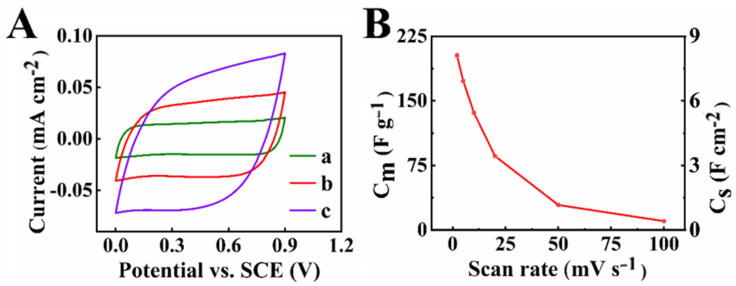
(**A**) CVs at scan rates of (a) 2, (b) 5, and (c) 10 mV s^−1^ and (**B**) capacitance calculated from CV data versus scan rate.

Cyclic voltammetry (CV) studies of the composite electrodes showed nearly rectangular CVs (Figure 5), and the highest capacitance calculated from CV data at 2 mV s^−1^ was 8.10 F cm^−2^ (202.6 F g^−1^). Capacitance decreased with an increasing scan rate in a relatively wide scan-rate range.

The kinetic analysis of charge storage properties of the electrodes was performed using equation [40,41,42,43,44]:

*I* = aν^b^ (2)

where *I* is the current, ν is the scan rate, and a and b are parameters. Parameter b was found to be 0.73 (Supplementary information, Figure S1). According to the literature [40,41,42,43,44], b = 0.5 for a battery-type materials and b = 1 for double-layer capacitive materials. The electrodes with 0.5 < b < 1 combine battery and capacitive properties. The battery type behavior is dominant for 0.5 < b < 0.8. Therefore, the tested electrode showed a dominant battery-type charging mechanism with a contribution of double-layer capacitance.

**Figure 6:**
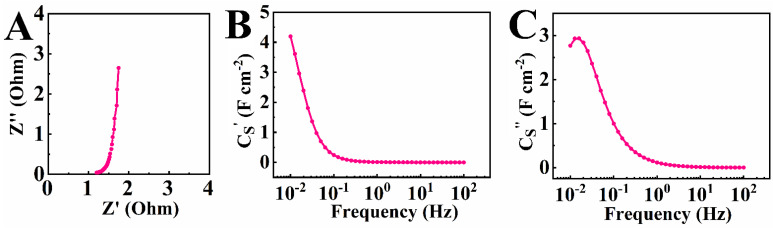
(**A**) Real (Z′) and imaginary Z″ components of complex impedance Z presented in a Nyquist plot; (**B**) real (Cs′) and (**C**) imaginary (Cs″) components of complex differential capacitance.

Electrochemical impedance spectroscopy (EIS) data showed relatively low impedance (Figure 6), and the Nyquist plot was a nearly vertical line, indicating good capacitive behavior. The highest real part of capacitance was 4.19 F cm^−2^ at a frequency of 10 mHz. The real part of capacitance decreased with frequency, and the frequency dependences of the components of complex capacitance showed relaxation-type dispersions.

**Figure 7:**
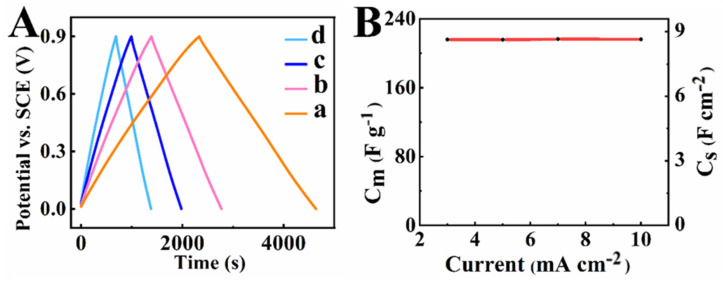
(**A**) GCD curves at current densities of (a) 3, (b) 5, (c) 7, and (d) 10 mA cm^−2^; (**B**) capacitance obtained from the GCD data versus current density.

**Figure 8:**
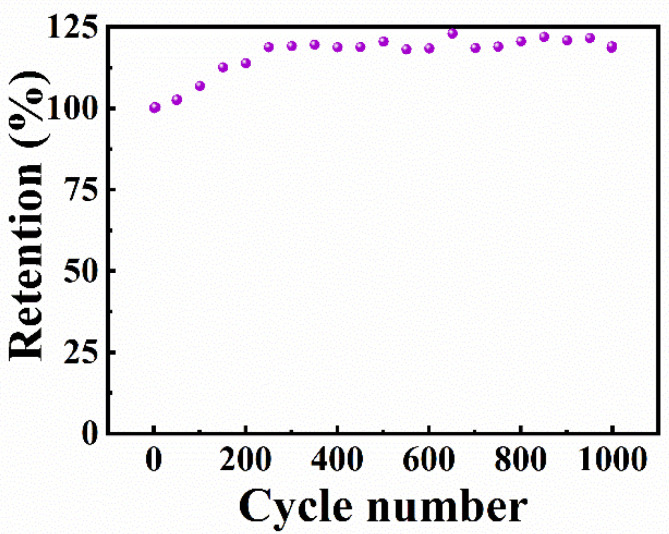
Capacitance retention versus cycle number obtained from CV data at a scan rate of 50 mV s^−1^.

The galvanostatic charge–discharge (GCD) curves (Figure 7) were of nearly triangular shapes, indicating good capacitive properties of the electrodes. The capacitance, calculated from GCD data at a current density of 3 mA cm^−2^, was 8.65 F cm^−2^ (216.16 F g^−1^). It was practically independent on the current density in the range of 3-10 mA cm^−2^. Investigations also revealed variations in capacitance during cycling. The capacitance (Figure 8) showed an initial increase by 18% during the first 250 cycles and then remained nearly constant. Similar capacitance increases were observed in previous investigations [35] and it was attributed to changes in the electrode’s microstructure during initial cycling.

The results of this investigation indicated that RL are promising biosurfactants for the fabrication of composite MnO_2_-MWCNT electrodes. It should be noted that many commercial surfactants can disperse only one type of material, such as inorganic oxides or carbon materials. In contrast, RL can be used as co-dispersants for MnO_2_ and MWCNT. The ability to co-disperse MnO_2_ and MWCNT was critically important for their efficient mixing, which allowed for enhanced electrode performances.

It is known [45] that MWCNT exhibits very low specific capacitance, typically around 20–40 F g^−1^. The use of MWCNT as a conductive additive is critically important, because MnO_2_ electrodes prepared without conductive additives showed [33] a capacitance of 22 F g^−1^ at the same active mass loading, which is significantly lower than the theoretical value of 1370 F g^−1^. The capacitance of MWCNT comprises double-layer capacitance, which is significantly lower than the redox-type capacitance of MnO_2_. Taking into account the small MWCNT content in the composite and the low capacitance of MWCNT, the high capacitance achieved in this investigation resulted from pseudocapacitive properties of MnO_2_. Many different materials are currently under investigation for energy storage in supercapacitors. MnO_2_ is one of the best materials for cathodes of supercapacitors due to large potential windows, nearly ideal pseudocapacitive behavior, high capacitance, and low costs. Capacitive behavior is strongly influenced by the active mass of the electrodes. Gravimetric capacitance decreases drastically with increasing active mass [32].

A recent comprehensive review [32] summarized the literature data for electrodes with high active mass loadings. Many investigations reported the fabrication of electrodes with an active mass of 10–35 mg cm^−2^ and revealed that it achieved significantly lower areal capacitances [32]. The areal capacitance obtained in this investigation is higher than the capacitances reported for MnO_2_ electrodes with the same active mass of 40 mg cm^−2^ [32]. Moreover, the areal and mass-normalized capacitances obtained in our investigation are higher than corresponding capacitances for MnO_2_ electrodes [32] with an active mass of 40–55 cm^−2^. Therefore, the method developed in this investigation allowed enhanced material performance at a high active mass. This method is very simple compared to other techniques, such as liquid–liquid extraction and electrostatic or Schiff base reaction heterocoagulation techniques [32]. It offers additional benefits of using a biocompatible RL biosurfactant.

## 3. Materials and Methods

RL, KMnO_4_, ethanol, Na_2_SO_4_, poly(vinyl butyral) (PVB, MilliporeSigma, Oakville, ON, Canada), and multiwalled carbon nanotubes (MWCNT, ID 4 nm, OD 13 nm, length 1–2 μm, Bayer, Leverkusen, Germany) were used as the starting materials. The as-received MWCNT formed large agglomerates with a typical diameter of 0.5 mm. MnO_2_ nanoparticles were prepared by a chemical precipitation method [46]. In this approach, 1.6 g KMnO_4_ was dissolved in 50 mL of water, and 25 mL of ethanol was added dropwise. Stirring was performed during 2 h. The reduction in KMnO_4_ with ethanol resulted in the formation of MnO_2_. After the addition of 0.2 g RL, the suspensions of MnO_2_ particles were ultrasonicated for 30 min and then MWCNTs were added to achieve a mass ratio of MnO_2_:MWCNT = 4:1. The suspension was ultrasonicated again for 30 min. After washing, filtration, and drying, the obtained mixtures were used for the fabrication of slurries in ethanol for the impregnation of commercial Ni foam (Vale, Toronto, ON, Canada) current collectors. The mass ratio of MnO_2_, MWCNT, and PVB binder was 80:20:3. The mass loading of the obtained electrodes after drying was 40 mg cm^−2^. For sedimentation tests, MnO_2_ was prepared without RL and MWCNT. MnO_2_ was dried in an oven at 60 °C for 24 h. The mass of RL, MnO_2_, and MWCNT was measured using Mettler Toledo XSR104 Excellence Analytical Balance (Mississauga, ON, Canada). RLs were dissolved in water to form 0.5 gL^−1^ solutions and required a certain amount of dried MnO_2_, or MWCNTs were added to achieve a mass ratio of RL:MnO_2_ = 0.2 or RL:MWCNT = 0.2, respectively. Both suspensions were ultrasonicated for 30 min before the sedimentation test.

Electron microscopy studies were performed using a JEOL SEM (scanning electron microscope, JEOL, JSM-7000F, IXRF, Inc., Austin, TX, USA). X-ray diffraction (XRD) analysis (diffractometer Bruker D8, Bruker, Billerica, MA, USA) was performed using Cu-Kα radiation at a rate of 0.01 degrees per second.

Electrochemical studies were performed in aqueous 0.5 M Na_2_SO_4_ electrolyte using PARSTAT 2273 (Ametek, Berwyn, the United States) potentiostat for cyclic voltammetry (CV) and electrochemical impedance spectroscopy (EIS), and the BioLogic VMP 300 (Biologic, Seyssinet-Pariset, France) potentiostat was used for galvanostatic charge–discharge (GCD) investigations. Testing was performed using a 3-electrode electrochemical cell containing a working electrode (impregnated Ni foam), counter-electrode (Pt mesh), and a reference electrode (SCE, saturated calomel electrode). The kinetic analysis of electrode performance was performed as described in [40,41,42,43,44] and presented in Supplementary Materials. The capacitive properties of electrode material were presented in gravimetric (C_m_, Fg^−1^) and areal (C_S_, F cm^−2^) capacitance forms. Capacitances C_m_ and C_S_ were calculated from CV, EIS, and GCD data as it was described in reference [32]. The capacitances calculated from CV and GCD data represented integral capacitances measured in a voltage window of 0–0.9 V versus SCE. The capacitances calculated from EIS data represented differential capacitances measured at an open-circuit potential at a voltage amplitude of 5 mV.

Capacitance was calculated from cyclic voltammetry (CV) data:

(3) $$C=\frac{\Delta Q}{\Delta U}=\frac{\left|\int_{0}^{t\left(U_{\max}\right)}I\mathrm{dt}\left|+\right|\int_{t\left(U_{\max}\right)}^{0}I\mathrm{dt}\right|}{2U_{\max}}$$

where ΔQ denotes charge, *I* denotes current, and ΔU denotes the potential range from chronopotentiometry data.

C = *I*Δt/ΔU (4)

Differential complex capacitance C*(ω) = C′(ω) − *i*C″(ω) was calculated at different frequencies (ω) from the complex impedance Z*(ω) = Z′(ω) + *i* Z″(ω) data.

(5) $$C^{\prime }\left(\omega \right)=\frac{-Z^{\prime \prime }\left(\omega \right)}{\omega {\left|Z\left(\omega \right)\right|}^{2}}$$

(6) $$C^{\prime \prime }\left(\omega \right)=\frac{Z^{\prime }\left(\omega \right)}{\omega {\left|Z\left(\omega \right)\right|}^{2}}$$

## 4. Conclusions

RLs are promising co-dispersants for MnO_2_ particles and MWCNT. The efficient co-dispersion of MnO_2_ and MWCNT facilitated their efficient mixing and allowed for the fabrication of electrodes with enhanced capacitances. The highest capacitance obtained from CV data at a scan rate of 2 mV s^−1^ was 8.10 F cm^−2^ (202.6 F g^−1^). The highest capacitance obtained from GCD data at a current density of 3 mA cm^−2^ was 8.65 F cm^−2^ (216.16 F g^−1^). The obtained capacitances are higher than the capacitances of MnO_2_-based electrodes of the same active mass reported in the literature. The approach developed in this investigation is simple compared to the other techniques used for the fabrication of electrodes with high active mass. It offers advantages in using a biocompatible RL biosurfactant.

## Data Availability

The data presented in this study are available in the following: “Application of rhamnolipids as dispersing agents for the fabrication of composite MnO_2_-carbon nanotube electrodes for supercapacitors”.

## Supplementary Materials

The following are available online, Figure S1: Current (i) versus scan rate (ν) dependence in a logarithmic scale used for the calculation of parameter b for electrodes from the equation i = aν^b^.

## Sample Availability

Samples of MnO_2_-MWCNT are available from the authors.
