# Supplementary material for: Application of Rhamnolipids as Dispersing Agents for the Fabrication of Composite MnO2-Carbon Nanotube Electrodes for Supercapacitors
Source: Molecules. 2022 Mar 3;27(5):1659. doi: 10.3390/molecules27051659 (PMC8911650; doi:10.3390/molecules27051659)
Supplement: Supplementary file 1 [file molecules-27-01659-s001.zip › molecules-1592839-supplementary.pdf]

molecules

Supplementary information

Application of Rhamnolipids as Dispersing Agents for the Fabrication of Composite MnO_2-Carbon Nanotube Electrodes for Supercapacitors

Wenjuan Yang, Wenyu Liang and Igor Zhitomirsky *

Department of Materials Science and Engineering, McMaster University, Hamilton, ON L8S4L7, Canada; yangw48@mcmaster.ca (W.Y.); liangw26@mcmaster.ca (W.L.)

* Correspondence: zhitom@mcmaster.ca

Citation: Yang, W.; Liang, W.; Zhitomirsky, I. Application of Rhamnolipids as Dispersing Agents for the Fabrication of Composite MnO_2-Carbon Nanotube Electrodes for Supercapacitors. *Molecules* **2022**, *27*, 1659. https://doi.org/10.3390/molecules27051659

Academic Editor: Tao Wang

Received: 26 January 2022

Accepted: 1 March 2022

Published: 3 March 2022

Publisher's Note: MDPI stays neutral with regard to jurisdictional claims in published maps and institutional affiliations.

Copyright: © 2022 by the authors. Licensee MDPI, Basel, Switzerland. This article is an open access article distributed under the terms and conditions of the Creative Commons Attribution (CC BY) license (https://creativecommons.org/licenses/by/4.0/).

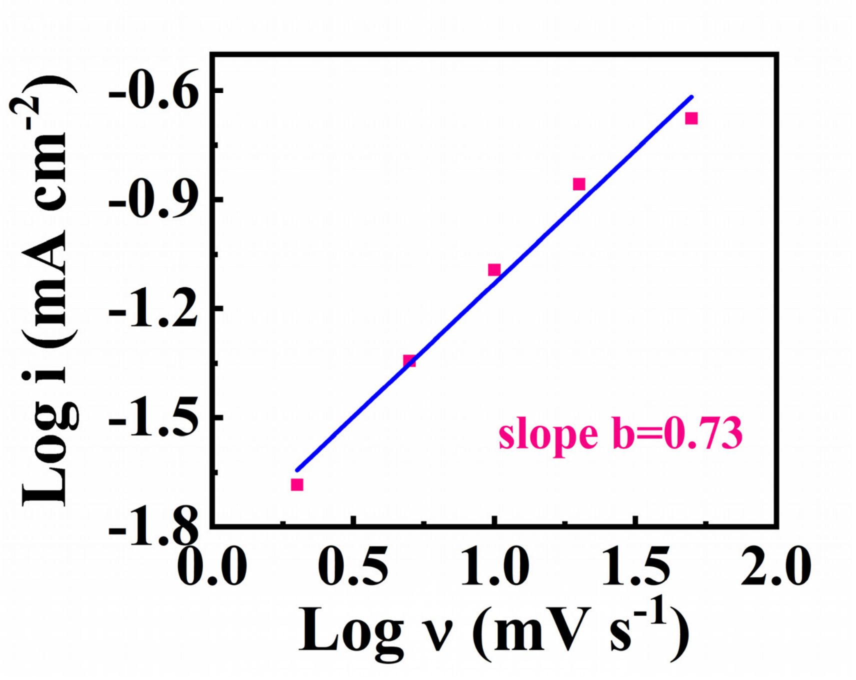

Figure S1. Current (i) versus scan rate (ν) dependence in a logarithmic scale used for the calculation of parameter b for electrodes from the equation $i = av^b$.
